# The combination of preoperative fibrinogen and neutrophil-lymphocyte ratio is a predictive prognostic factor in esophagogastric junction and upper gastric cancer

**DOI:** 10.7150/jca.31162

**Published:** 2019-08-29

**Authors:** Xiliang Cong, Sen Li, Yongle Zhang, Ziyu Zhu, Yimin Wang, Shubin Song, Yan Ma, Rui Xie, Yingwei Xue

**Affiliations:** 1 Department of Gastrointestinal Surgery, Harbin Medical University Cancer Hospital, Harbin, China; 2 Department of General Surgery, The Affiliated Cancer Hospital of Zhengzhou University, Zhengzhou, China; 3 Department of Digestive Internal Medicine & Photodynamic Therapy Center, Harbin Medical University Cancer Hospital, Harbin, China

**Keywords:** gastric cancer, adenocarcinoma of esophagogastric junction, neutrophil-lymphocyte ratio, fibrinogen, prognosis.

## Abstract

**Objective**: Cancer-associated systemic inflammation response and hyperfibrinogenemia play crucial roles in cancer progression and prognosis. In this study, we assessed the clinical value of the preoperative fibrinogen and the neutrophil-lymphocyte ratio (NLR) in patients with adenocarcinoma of the esophagogastric junction (AEG) and upper gastric cancer (UGC).

**Methods**: Patients with AEG or UGC who underwent curative surgery were divided into a training set (n=161) and a validation set (n=195). Univariate and multivariate Cox analyses were performed to evaluate the prognostic indicators for overall survival (OS). The optimization cut-off values for fibrinogen and the NLR were 3.09g/L and 1.84, respectively. The combination of fibrinogen and NLR (F-NLR) was 2 for patients with high fibrinogen (≥3.09g/L) and elevated NLR (≥1.84), whereas those with one or neither were indexed as 1 or 0, respectively.

**Results**: F-NLR was identified as an independent prognostic indicator for OS in the training set (*P*=0.007) which was confirmed in the validation set (*P*=0.003). In the subgroup analyses, the prognostic significance of F-NLR was still maintained for stages I-II (*P* = 0.030 in the training set; and *P* =0.020 in the validation set) and III (*P* = 0.001 in the training set; and *P* <0.001 in the validation set).Notably, among patients with F-NLR 2 could benefit from adjuvant chemotherapy compared with those with F-NLR 0-1 (*P* = 0.020 in the training set; and *P* =0.005 in the validation set).

**Conclusions**: The preoperative F-NLR score is an independent prognosis indicator for patients with AEG and UGC. And it may help clinicians to identify those patients who at high prognostic risk and will benefit from planning individualized treatment strategies.

## Introduction

Gastric cancer is one of the common aggressive malignant tumors, with a high ratio of tumor recurrence and mortality 1, 2. According to the position of the main tumor, gastric cancer is classified into upper, middle or lower third cancers. Although the pathological characteristics are similar, the position of tumor has an influence on the postoperative quality of life and survival of gastric cancer patients. Adenocarcinoma of the esophagogastric junction (AEG), is a representative malignancy located between the esophagus and stomach, and was originally characterized by Siewert 3. It was well-known to have unique clinicopathological features and biological behavior. In recent decades, the incidence rate of AEG gradually rose globally, particularly in the western countries 4, 5. AEG and upper gastric cancer (UGC) patients undergo surgery, a total gastrectomy is usually required. Recent researches have reported that distal gastrectomy provide a better long-term outcome for distal gastric cancer patients compared with total gastrectomy 6, 7. Besides some studies have indicated that the prognosis for AEG is worse compare with distal gastric cancer patients 8. Therefore, it is important to search suitable clinical prognostic factors to supply more accurate and precise evaluates of survival, extremely important in high-fatality malignancies such as AEG. This can both enhance outcomes and decrease costs by better choosing patients for eligible treatment 9.

Cancer-related systemic inflammatory response plays an important role in the progression and outcome of tumors 10, 11. We previously reported that systemic immune-inflammation score SII (SII=N×P∕L), which was based on neutrophil (N), platelet (P) and lymphocyte (L) counts, had been demonstrated to be a predictive prognostic indicator in patients with advanced gastric cancer undergoing neoadjuvant chemotherapy 12. Also, several common inflammation-based prognostic scoring systems, such as the neutrophil-lymphocyte ratio (NLR), platelet-lymphocyte ratio (PLR) and lymphocyte-monocyte ratio (LMR) have been reported to have prognostic value in various cancers 13-16. In addition, the hemostatic also plays a key role in cancer progression and metastasis 17, 18. Liver-produced fibrinogen is a key factor in the hemostatic cascade. Recent studies have confirmed that fibrinogen correlates with cancer progression, poor response to chemotherapy and adverse prognostic outcome in various malignancies 19-21. Recently, several researches analyzed a new scoring system, that is, combining preoperative fibrinogen and the NLR (F-NLR). F-NLR has been demonstrated to be a significant prognostic marker in several types of cancers, such as non-small cell lung cancer, esophageal squamous cell carcinoma and gastric cancer 22-24.

Therefore, the current study aimed to evaluate the prognostic value of F-NLR in patients with AEG and UGC.

## Materials and Methods

### Patients

Two independent cohorts comprising 356 consecutive patients with AEG or UGC who underwent curative surgery were enrolled into the present retrospective study from Harbin Medical University Cancer Hospital. The training set that included 161 consecutive patients was collected between 2007 and 2011, and the validation set that included 195 consecutive patients was collected between 2012 and 2016 with the same enrolment criteria. The patients enrolled this analysis met the following inclusion criteria: 1) pathologically confirmed adenocarcinoma, 2) no neoadjuvant chemotherapy and/or radiotherapy before operation, 3) complete clinicopathologic parameters and outcome. The major exclusion criteria included: 1) multiple primary malignances, 2) hematological disease, bone marrow disease and autoimmune disease, 3) active infection or other inflammatory disease for nearly 1 month before surgery, 4) death within perioperative period. The Siewert classification was introduced to about tumor position 3. According to previous published reports, 25, 26 AEG was well- defined as Siewert type I, II, and III tumors and tumors with the center was situate exceed 5 cm below the gastroesophageal junction within the upper one third stomach as UGC. Clinicopathological parameters and laboratory inspections of the patients were acquired from the medical records, including sex, age, tumor size, tumor location, histologic differentiation, surgical procedure, pTNM stage and blood cell count. The pTNM stage was according to the 8th TNM classification of American Joint Committee on Cancer (AJCC) staging manual. Permission for this retrospective cohort research was approved by the ethics committee of Harbin Medical University Cancer Hospital.

### Evaluation of prognostic scores

Hematological laboratory measurements including neutrophil count, lymphocyte count, monocyte count and fibrinogen concentrations, were extracted from the daily blood test administered in the week before surgery. According to the Youden index by Receiver operating characteristic (ROC) curve, the most appropriate cutoff threshold was found as 3.09g/L for plasma fibrinogen and 1.84 for NLR in the training cohort, and was then applied to the validation cohort. For these values, an area under the curve (AUC) as 0.650( 95%CI: 0.565-0.735) and 0.615(95% CI:0.527-0.702), respectively. Similarly, the optimal cutoff values of 110, 451 and 3.25 for PLR, SII and LMR also determined by ROC curve. Based on these cut-off values, the F-NLR score was classified as follows: F-NLR score of 2 [both a hyperfibrinogenemia (≥3.09g/L) and high NLR (≥1.84)], 1 [either hyperfibrinogenemia (≥3.09g/L) or high NLR (≥1.84)], 0 [neither hyperfibrinogenemia nor high NLR].

### Statistical analysis

Statistical analysis was done using SPSS software version 22 (IBM, Armonk, New York, USA). A two-tailed chi-squared test and Spearman-rho test was used to evaluate differences in categorical variables. Differences between the overall survival (OS) generated by the Kaplan-Meier curves were decided using the log-rank test. OS was defined as the time in months between the date of surgery and the date of death or last follow-up. Univariate and multivariate analyses were carried out by Cox regression models to clarify the independent prognostic factors. Prognostic value and accuracy of the F-NLR prognostic models was assessed by receiver operating characteristic (ROC) analysis. All *P* values were quoted two-sided, and a *P* value of <0.05 was considered to represent statistically significant.

## Results

### Patient characteristics

Baseline characteristics clinicopathological of patients are illustrated in Table 1. In the training cohort, 161 patients (126 men [78.3%] and 35 women [21.7%]) were included. The median age was 61 (range 34-76) years. The median and mean follow-up duration were 43.7 and 52.6 months, respectively. In the validation cohort, 195 patients (154 men [79.0%] and 41 women [21.0%]) were included. The median age was 62 (range 32-76) years. The median and mean follow-up duration were 49.8 and 54.7 months, respectively.

Due to the limitation of patient number, we combined the training and the validation cohorts to the combined cohort. The relationship between F-NLR and clinicopathological variables is shown in Supplementary Table S1. There was significant correlation of F-NLR with tumor size, PLR, LMR and SII in all 3 cohorts.

### Prognostic analysis based on plasma fibrinogen, NLR or F-NLR

We conducted the Kaplan-Meier analysis and log-rank test to determine the survival differences between the groups categorized by fibrinogen, NLR or F-NLR. Patients with hyperfibrinogenemia had a much worse OS than those with low fibrinogen (*P*<0.001 in all 3 sets; Figure S1A-C). Patients with increased NLR had a poorer OS than those with low NLR (*P*=0.005 in the training set; *P*=0.014 in the validation set; *P*<0.001 in the combined set; Figure S1D-F, respectively). Furthermore, further analysis showed that plasma fibrinogen had a positive and significant correlation with NLR (Table S2).

**Figure 1:**
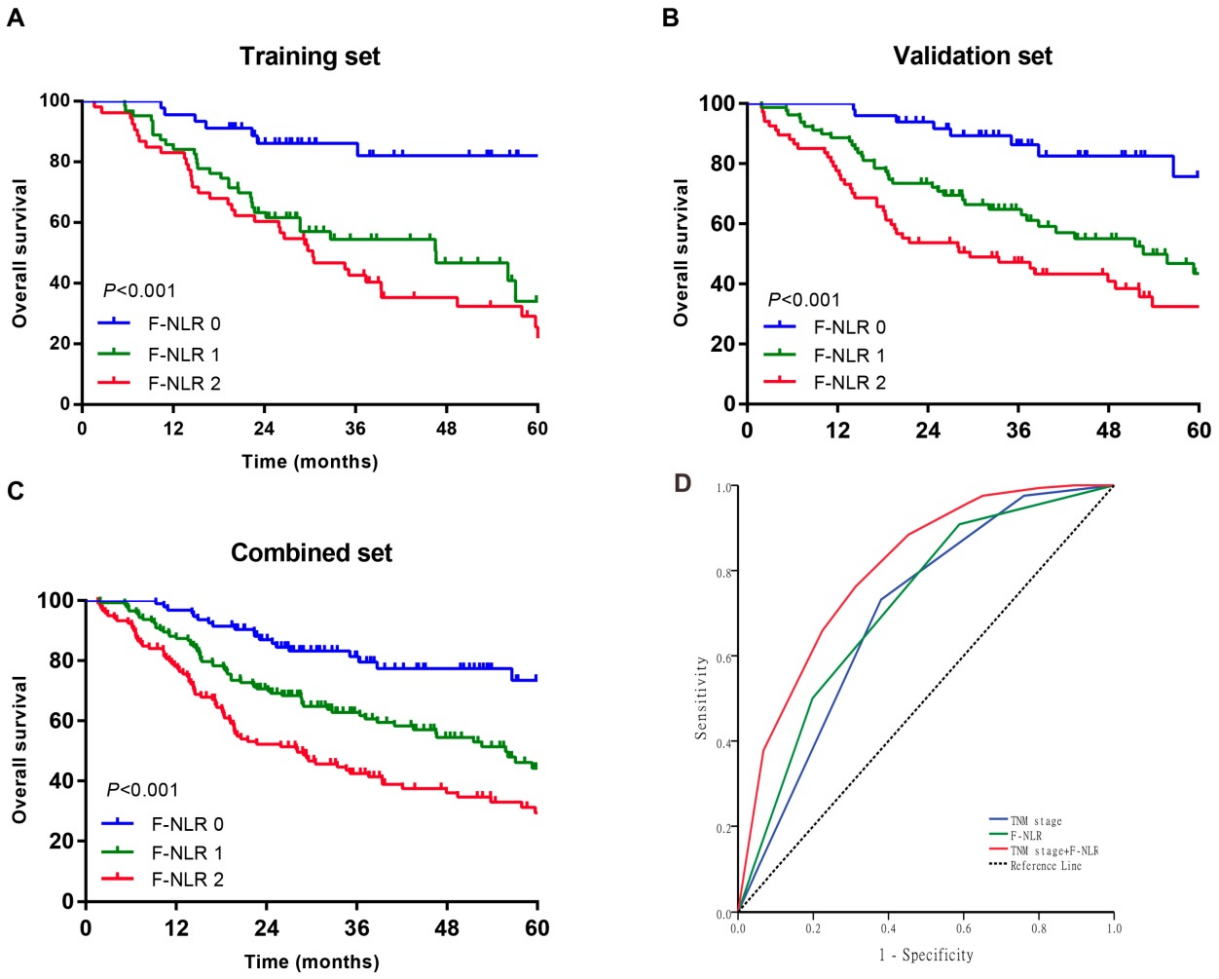
Survival curves of patients with AEG and UGC according to the combination of fibrinogen concentration and NLR (F-NLR). A-C, Overall survival (OS) of patients with F-NLR=0, F-NLR=1, and F-NLR=2 in the A, training set (n = 161, *P* <0.001); B, validation set (n = 195, *P* <0.001); and C, combined set (n = 356, *P* <0.001). D, Receiver operating characteristic of TNM stage (area under the curve [AUC] = 0.700) vs F-NLR (AUC = 0.717) vs TNM stage +F-NLR (AUC = 0.803)

**Figure 2:**
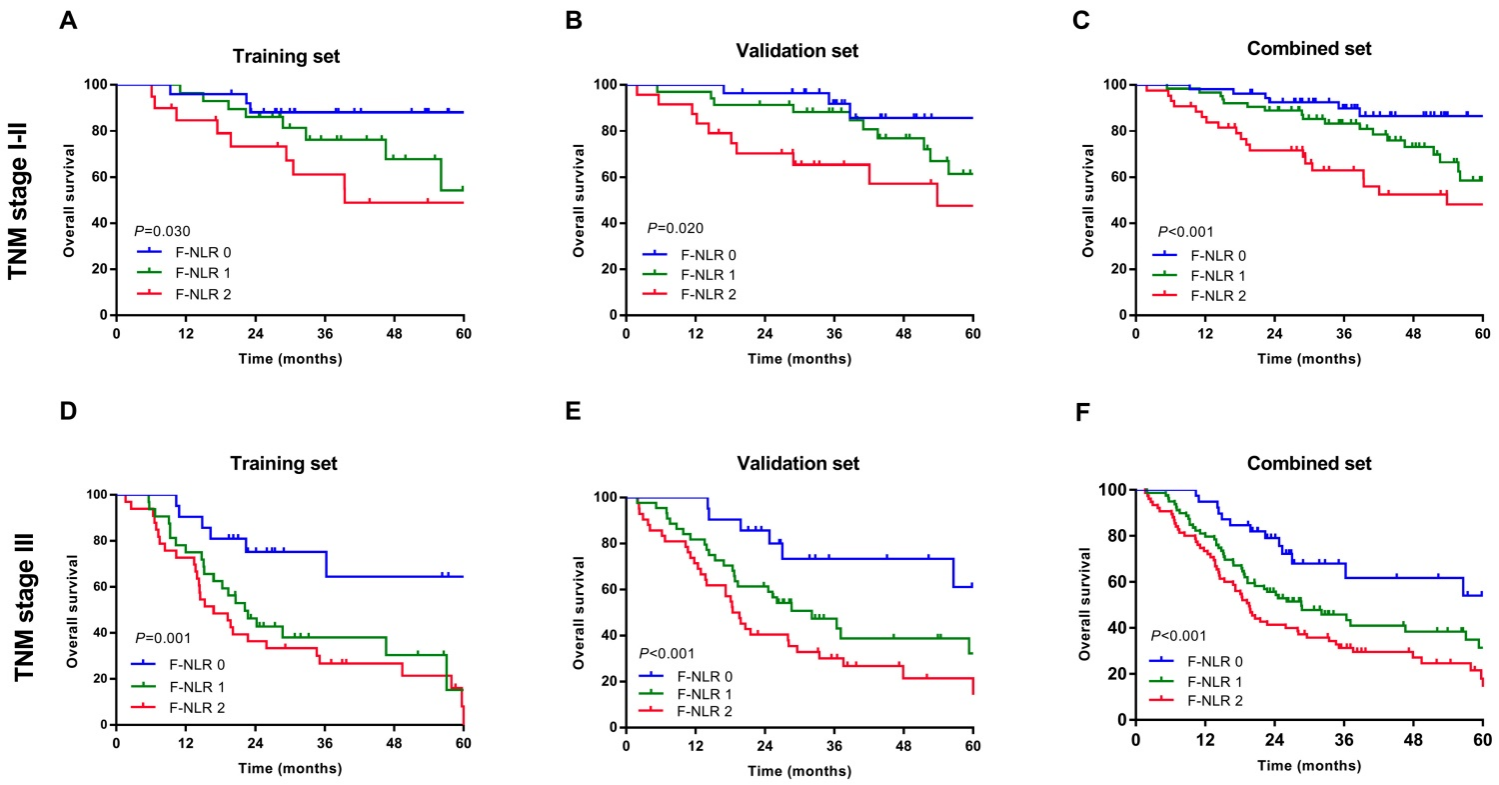
Survival curves based on the F-NLR of AEG and UGC patients (TNM stage I-III). A-C, Overall survival (OS) of patients with TNM stage I-II with F-NLR=0, F-NLR=1, and F-NLR=2 in the A, training set (*P* =0.030); B, validation set (*P* =0.020); and C, combined set (*P* <0.001). D-E, Overall survival (OS) of patients with TNM stage III with F-NLR=0, F-NLR=1, and F-NLR=2 in the A, training set (*P*=0.001); B, validation set (*P* <0.001); and C, combined set (*P* <0.001).

As shown in Figure 1A-C, patients with F-NLR 2 showed compromised OS compared to patients with F-NLR 0 or F-NLR 1 in the training (*P*< 0.001), validation (*P*< 0.001) and combined sets (*P*<0.001).When stratified by pathological TNM stages (I, II and III) were analyzed separately, the OS of patients with F-NLR 0 or F-NLR 1 were higher than those with F-NLR 2 in stages I-II (*P*=0.030 in the training set; *P*=0.020 in the validation set; *P*<0.001 in the combined set; Figure 2A-C, respectively) and stage III (*P*=0.001 in the training set; *P*<0.001 in the validation set; *P*<0.001 in the combined set; Figure 2D-F, respectively).

### Univariate and multivariate regression analyses

To identify the independent prognostic indexes for OS, we carried out univariate and multivariate analyses with a Cox proportional hazard model. As shown in Table 2, a high F-NLR score confirmed to be a significant negative prognostic factor in all 3 sets (*P*<0.001). In addition, tumor size (*P*<0.001 in all 3 sets), pathological TNM stages(*P*<0.001 in all 3 sets), PLR (*P*=0.007 in the training set; *P*<0.001 in the validation set; *P*<0.001 in the combined set, respectively), LMR (*P*=0.015 in the training set; *P*=0.007 in the validation set; *P*=0.004 in the combined set, respectively) and SII (*P*=0.014 in the training set; *P*<0.001 in the validation set; *P*<0.001 in the combined set, respectively) was also proved to be significantly associated with OS. Moreover, Age (*P*=0.023) was identified to be significantly correlated with OS in the training set, as was surgical procedure in the validation set (*P*=0.049) and in the combined set (*P*=0.043).These indicators were then included into the multivariate Cox proportional hazards model, and we found that only F-NLR (*P*=0.007; *P*=0.003; *P*=0.002; Table 3, respectively) and pathological TNM stages (*P*<0.001) were independent prognostic factors for OS in all 3 sets. Tumor size was independent prognostic factor in the validation set (*P*=0.024) and in the combined set (*P*=0.042), but not in the training set (*P*=0.425).

### Extension and accuracy of prognostic models with FNLR

Because of the distinctly prognostic value, we united F-NLR into the pathological TNM staging system to evaluate the practical application of F-NLR. ROC analysis was applied to assess the prognostic accuracy. As shown in Figure 1D, the AUC of pathological TNM stage alone was 0.700 (95% CI: 0.646-0.754) as compared with 0.717 (95% CI: 0.664-0.770) for the F-NLR. When F-NLR were added into the pTNM staging system, the AUC was elevated to 0.803 (95% CI: 0.758-0.848).

### F-NLR as a predictor for the choice of postoperative treatment patter in AEG and UGC patients

**Figure 3:**
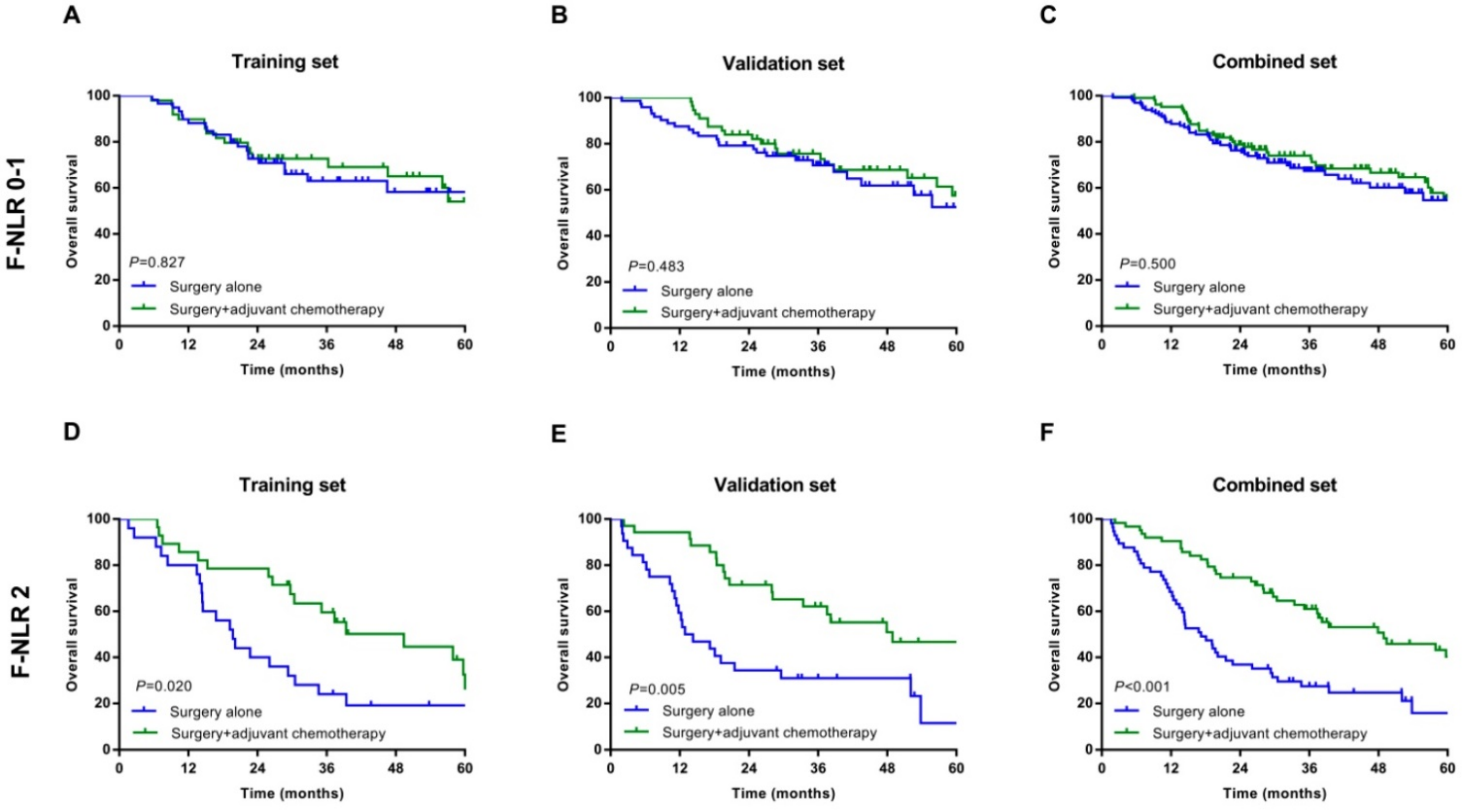
Relationship between F-NLR and benefit from adjuvant chemotherapy in patents with F-NLR 0-1 and F-NLR 2. Patients with F-NLR 0-1 in the A, training set (*P*=0.827); B, validation set (*P*=0.483); and C, combined set (*P*=0.500). Patients with F-NLR 2 in the D, training (*P*=0.020) set; E, validation set (*P*=0.005); and F, combined set (*P*<0.001).

In the subgroup analysis, we assessed the association between postoperative adjuvant chemotherapy and OS. In patients with F-NLR 0/1 could not benefit from adjuvant chemotherapy in training, validation and combined sets (*P*=0.827, *P*=0.483, *P*=0.500, Figure 3A-C, respectively). However, those patients with F-NLR 2 could benefit greatly from adjuvant chemotherapy (*P*=0.020, *P*=0.005, *P*<0.001, Figure 3D-F, respectively).

## Discussion

Although with the rapid developments in surgical techniques and adjuvant treatments, the median survival of gastrointestinal malignancies remains unsatisfactory 27. A proper prognostic factor can allow patients with tumors to have an appropriate risk classification and allow for the adequate treatment to be assigned. Cancer progression and prognosis are not just determined by clinical and pathological features of the tumor. Personalized factors can also play a central role in estimate of survival. In our current retrospective study, we investigated the prognostic value of F-NLR score and the relationship between F-NLR and clinicopathological features in the patients with pTNM stages I-III AEG and UGC.

Inflammation and immune cells are essential components of the tumor microenvironments. By creating a favorable microenvironment and inhibiting anti-tumor immunity, systemic inflammatory responses of tumor cells are important in tumor growth, progression and metastasis 28. A growing of evidence suggests that systemic inflammation responses are key prognostic indicators 29, 30. The systemic inflammatory response disrupts balance of circulating white blood cell components 31. Thus, it affects the number of neutrophils and lymphocytes in leukocyte during cancer progression. The neutrophil- lymphocyte ratio (NLR) has been recognized as a representative prognostic indicator in various malignancies, including gastric cancer 32-34.

In addition, more and more studies have demonstrated that the association between haemostatic system and cancer progression in recent years 17, 18. Increasing evidence performs that the activation of the haemostatic cascade plays a crucial pathophysiological role in tumor aggressiveness 35. Fibrinogen is a main acute-phase protein and as an important component of the haemostatic system, has been shown to be a necessary regulator of the systemic inflammatory state and malignancy progression 36.It may mediate the original adhesion of white blood cells to endothelial cells and the release of pro-inflammatory cytokines, thus induce cancer cell proliferation and progression 37. Hyperfibrinogenemia has been confirmed to be a significant prognostic predictor with tumor progression and poor response to chemotherapy in various malignancies 19-21.

Therefore, the combination serum fibrinogen and NLR (F-NLR) provides a good prognostic marker for cancer patients. Fibrinogen alone or NLR may have a limited effect on tumor progression. F-NLR increases the adverse effects of fibrinogen and NLR, ultimately increasing the predictive significance of cancer patients. Recently, the prognostic value of F-NLR was further demonstrated in various studies. Huang et al 22, proved that preoperative F-NLR scores can be a valuable prognostic marker for patients with early resectable non-small cell lung cancer. Kijima et al 23, reported that the F-NLR score is promising to be a predictor of therapeutic effects and prognosis in patients undergoing esophagectomy for advanced esophageal squamous cell carcinoma. Liu et al 24, demonstrated that F-NLR score independently predicts outcomes of patients with gastric cancer underwent curative surgery, consistent with the findings of our study. In the current study, we proved F-NLR as an independent prognostic factor for OS in AEG and UGC patients and integrated it into the pathological TNM staging system to improve its prognostic value. When the patients with different pathological TNM stages were analyzed separately, the F-NLR score still displayed potential prognostic value. Furthermore, we also found that the high-risk patients according to F-NLR 2 may benefit from postoperative adjuvant chemotherapy. Thus, to evaluate the pathological situation of tumor progression, preoperative FNLR levels counted from blood samples should be assessed. The fact that F-NLR score can be obtained from the routine blood sample makes it practical and inexpensive.

This study had several shortages. First of all, this study was a single institute, retrospective analysis and could not avoid the bias in population selection. Second, although we restricted some possible mixed factors, the hematologic cell counts can be influenced by several factors. Third, we were short of the follow-up information for disease-free survival, and our conclusions may be reinforced by using other methods of survival.

## Conclusion

The preoperative F-NLR score is an independent predictor of survival in patients who underwent curative surgery for AEG and UGC. As it is objectively measured and daily available, which may be a useful clinical biomarker for identifying patients at high prognostic risk and planning individualized treatment strategies for patients with AEG and UGC.

## Supplementary Material

Supplementary figure and tables.

## Figures and Tables

**Table 1:** Clinicopathologic characteristics of AEG and UGC patients.

	Training set		Validation set		Combined set	
Characteristics	Number	%	Number	%	Number	%
All patients	161	100	195	100	356	100
Sex						
Female	35	21.7	41	21.0	76	21.3
Male	126	78.3	154	79.0	280	78.7
Age(years)						
<60	72	44.7	73	37.4	145	40.7
≥60	89	55.3	122	62.6	211	59.3
Tumor size(cm)						
<5	74	46.0	88	45.1	162	45.5
≥5	87	54.0	107	54.9	194	54.5
Location						
UGC	95	59.0	132	67.7	227	63.8
AEG	66	41.0	63	32.3	129	36.2
Differentiation						
Well/Moderate	38	23.6	50	25.6	88	24.7
Poor	123	76.4	145	74.4	268	75.3
Surgical procedure						
Proxima gastrectomy	92	57.1	124	63.6	216	60.7
Total gastrectomy	69	42.9	71	36.4	140	39.3
NLR						
<1.84	77	47.8	83	42.6	160	44.9
≥1.84	84	52.2	112	57.4	196	55.1
Fibrinogen (g/L)						
<3.09	76	47.2	94	48.2	170	47.8
≥3.09	85	52.8	101	51.8	186	52.2
PLR						
<110	76	47.2	78	40.0	154	43.3
≥110	85	52.8	117	60.0	202	56.7
LMR						
<3.25	53	32.9	47	24.1	100	28.1
≥3.25	108	67.1	148	75.9	256	71.9
SII						
<451	85	52.8	102	52.3	187	52.5
≥451	76	47.2	93	47.7	169	47.5
pTNM stage						
I	19	11.8	30	15.4	49	13.8
II	56	34.8	58	29.7	114	32.0
III	86	53.4	107	54.9	193	54.2
F-NLR						
0	45	28.0	49	25.1	94	26.4
1	63	39.1	79	40.5	142	39.9
2	53	32.9	67	34.4	120	33.7
Adjuvant chemotherapy						
Yes	77	47.8	91	46.7	168	47.2
No	84	52.2	104	53.3	188	52.8

AEG: adenocarcinoma of esophagogastric junction; UGC: upper gastric cancer; NLR: neutrophil-lymphocyte ratio; PLR: platelet-lymphocyte ratio; LMR: lymphocyte-monocyte ratio; SII: (SII=N×P/L), which was based on neutrophil (N), platelet (P) and lymphocyte (L) counts; F-NLR: combination of fibrinogen concentration and neutrophil-lymphocyte ratio.

**Table 2:** Univariate Cox regression analyse for OS.

	Training set								Validation set								Combined set							
Characteristics	HR		95%CI				*P*		HR		95%CI				*P*		HR		95%CI				*P*	
Sex							0.463								0.367								0.775	
Female	1.00								1.00								1.00							
Male	0.812		0.467-1.415						1.284		0.746-2.208						1.058		0.719-1.557					
Age(years)							**0.023**								0.869								0.102	
<60	1.00								1.00								1.00							
≥60	1.733		1.080-2.780						1.037		0.671-1.603						1.305		0.948-1.795					
Tumor size(cm)							**0.001**								**<0.001**								**<0.001**	
<5	1.00								1.00								1.00							
≥5	2.247		1.376-3.670						3.111		1.941-4.988						2.681		1.909-3.764					
Location							0.927								0.088								0.083	
UGC	1.00								1.00								1.00							
AEG	1.022		0.646-1.615						1.461		0.945-2.260						1.320		0.965-1.807					
Differentiation							0.243								0.215								0.094	
Well/Moderate	1.00								1.00								1.00							
Poor	1.402		0.795-2.470						1.379		0.830-2.293						1.381		0.946-2.015					
Surgical procedure							0.415								**0.049**								**0.043**	
Proxima gastrectomy	1.00								1.00								1.00							
Total gastrectomy	1.207		0.768-1.898						1.527		1.002-2.326						1.374		1.010-1.870					
NLR							**0.006**								**0.015**								**<0.001**	
<1.84	1.00								1.00								1.00							
≥1.84	1.941		1.207-3.120						1.737		1.113-2.711						1.820		1.316-2.517					
Fibrinogen (g/L)							**<0.001**								**<0.001**								**<0.001**	
<3.09	1.00								1.00								1.00							
≥3.09	2.546		1.539-4.212						2.606		1.648-4.122						2.598		1.851-3.645					
PLR							**0.007**								**<0.001**								**<0.001**	
<110	1.00								1.00								1.00							
≥110	1.913		1.190-3.076						2.750		1.669 4.533						2.229		1.592-3.122					
LMR							**0.015**								**0.007**								**0.004**	
<3.25	1.00								1.00								1.00							
≥3.25	0.568		0.360-0.897						0.536		0.340-0.844						0.620		0.449-0.855					
SII							**0.014**								**<0.001**								**<0.001**	
<451	1.00								1.00								1.00							
≥451	1.771		1.122-2.795						2.272		1.478-3.492						2.020		1.478-2.762					
pTNM stage							**<0.001**								**<0.001**								**<0.001**	
I	1.00								1.00								1.00							
II	4.133		0.958-17.833				0.057		6.394		1.497-27.299				0.012		3.631		1.433-9.201				0.007	
III	12.370 2.969-51.540						0.001		15.255 3.728-62.423						<0.001		9.580		3.910-23.471				<0.001	
F-NLR							**<0.001**								**<0.001**								**<0.001**	
0	1.00								1.00								1.00							
1	4.029		1.772-9.163				0.001		3.252		1.511-6.998				0.003		2.506		1.502-4.182				<0.001	
2	5.604		2.502-12.554				<0.001		5.534		2.604-11.762				<0.001		4.116		2.496-6.785				<0.001	
Adjuvant chemotherapy							0.202								0.146								0.106	
Yes	1.00								1.00								1.00							
No	1.345		0.853-2.121						1.368		0.896-2.088						1.290		0.868-1.608					

OS: overall survival; HR: hazard ratio; CI: confidence interval; UGC: upper gastric cancer; AEG: adenocarcinoma of esophagogastric junction; NLR: neutrophil-lymphocyte ratio; PLR: platelet- lymphocyte ratio; LMR: lymphocyte-monocyte ratio; SII:(SII=N×P/L), which was based on neutrophil (N), platelet (P) and lymphocyte (L) counts; F-NLR: combination of fibrinogen concentration and neutrophil-lymphocyte ratio.

**Table 3:** Multivariate Cox regression analyse for OS.

	Training set								Validation set								Combined set							
Characteristics	HR		95%CI				*P*		HR		95%CI				*P*		HR		95%CI				*P*	
Age(years)							0.644																	
<60	1.00																							
≥60	1.129		0.675-1.886																					
Tumor size(cm)							0.425								**0.024**								**0.042**	
<5	1.00								1.00								1.00							
≥5	1.257		0.717-2.202						1.784		1.079-2.949						1.455		1.015-2.088					
Surgical procedure															0.959								0.730	
Proxima gastrectomy									1.00								1.00							
Total gastrectomy									1.012		0.650-1.574						1.057		0.772-1.447					
PLR							0.612								0.871								0.327	
<110	1.00								1.00								1.00							
≥110	1.160		0.654-2.058						1.050		0.580-1.900						1.238		0.807-1.900					
LMR							0.052								0.113								0.271	
<3.25	1.00								1.00								1.00							
≥3.25	0.600		0.359-1.005						0.674		0.414-1.098						0.820		0.576-1.167					
SII							0.320								0.715								0.863	
<451	1.00								1.00								1.00							
≥451	1.403		0.720-2.736						1.105		0.646-1.890						1.040		0.668-1.618					
pTNM stage							**<0.001**								**<0.001**								**<0.001**	
I	1.00								1.00								1.00							
II	3.682		0.834-16.250				0.085		5.045		1.155-22.038				0.031		2.774		1.079-7.134				0.034	
III	11.304 2.637-48.455						0.001		10.634 2.489-45.433						0.001		6.118		2.418-15.478				<0.001	
F-NLR							**0.007**								**0.003**								**0.002**	
0	1.00								1.00								1.00							
1	3.394		1.417-8.129				0.006		2.860		1.278-6.397				0.011		1.921		1.124-3.283				0.017	
2	4.591		1.776-11.866				0.002		4.432		1.880-10.450				0.001		2.764		1.559-4.900				0.001	

OS: overall survival; HR: hazard ratio; CI: confidence interval; UGC: upper gastric cancer; AEG: adenocarcinoma of esophagogastric junction; PLR: platelet-lymphocyte ratio; LMR: lymphocyte-monocyte ratio; SII:(SII=N×P/L), which was based on neutrophil (N), platelet (P) and lymphocyte (L) counts; F-NLR: combination of fibrinogen concentration and neutrophil-lymphocyte ratio.
