# Supplementary material for: The combination of preoperative fibrinogen and neutrophil-lymphocyte ratio is a predictive prognostic factor in esophagogastric junction and upper gastric cancer
Source: J Cancer. 2019 Aug 29;10(22):5518–26. doi: 10.7150/jca.31162 (PMC6775700; doi:10.7150/jca.31162)
Supplement: Supplementary file 1 — Supplementary figure and tables. [file jcav10p5518s1.pdf]

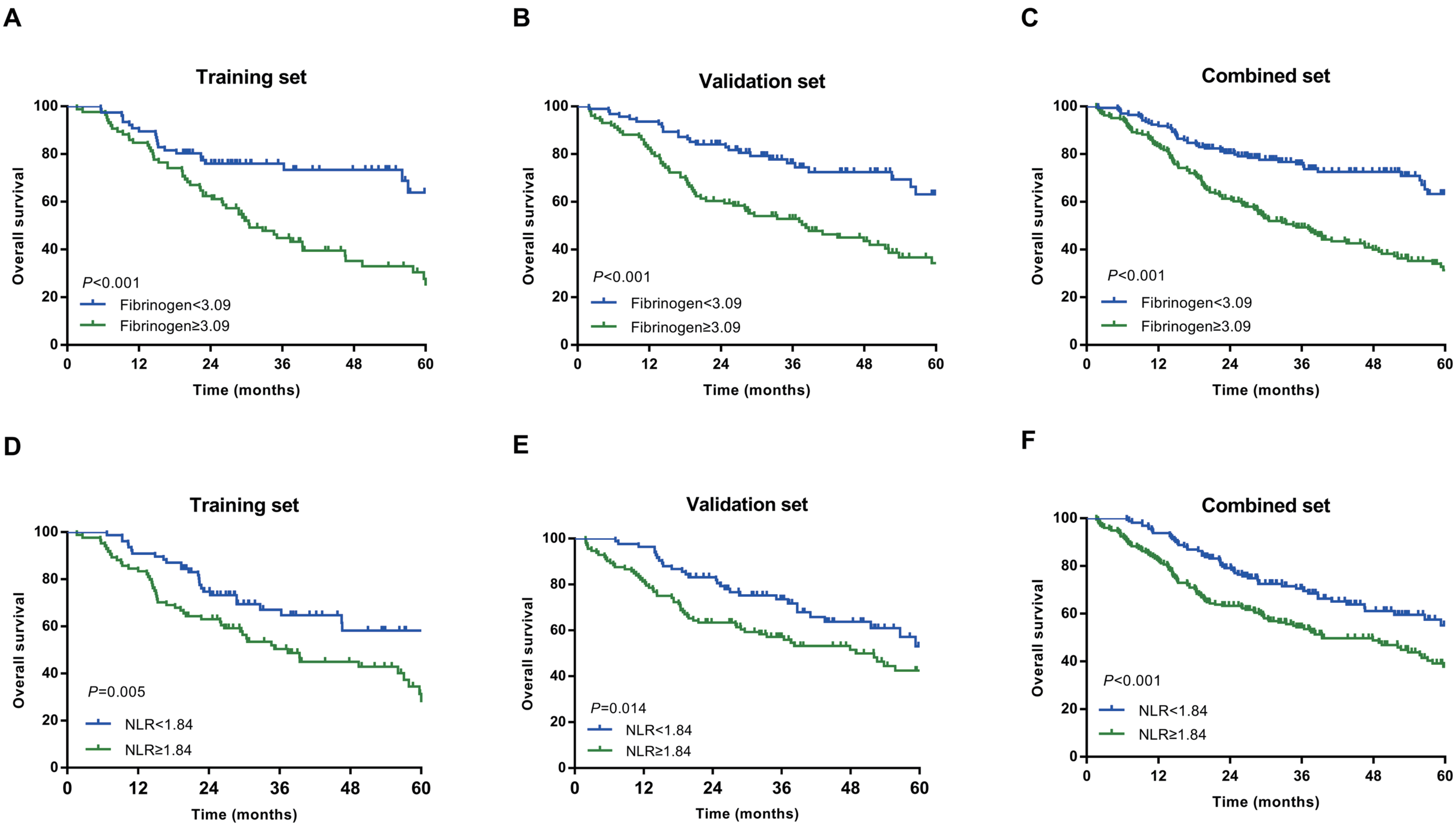
A
Training set
Overall survival
100
80
60
40
20
0
0
12
24
36
48
60
Time (months)
P<0.001
Fibrinogen<3.09
Fibrinogen≥3.09
B
Validation set
Overall survival
P<0.001
Fibrinogen<3.09
Fibrinogen≥3.09
Time (months)
C
Combined set
Overall survival
P<0.001
Fibrinogen<3.09
Fibrinogen≥3.09
Time (months)
D
Training set
Overall survival
P=0.005
NLR<1.84
NLR≥1.84
Time (months)
E
Validation set
Overall survival
P=0.014
NLR<1.84
NLR≥1.84
Time (months)
F
Combined set
Overall survival
P<0.001
NLR<1.84
NLR≥1.84
Time (months)

Figure S1. Survival curves of patients with AEG and UGC according to the combination of fibrinogen concentration and NLR (F-NLR). A-C, Overall survival (OS) of patients with fibrinogen<3.09g/L and fibrinogen≥3.09g/L in the A, training set (*P*<0.001); B, validation set (*P*<0.001); and C, combined set (*P*<0.001). D-F, Overall survival (OS) of patients with NLR<1.84 and NLR≥1.84 in the D, training set (*P*=0.005); E, validation set (*P*=0.014); and F, combined set (*P*<0.001).

Table S1. Correlation between preoperative F-NLR and clinicopathologic characteristics of patients with AEG and UGC.

		Training set				Validation set				Combined set			
**Characteristics**	**F-NLR score**	**0**	**1**	**2**	***P***	**0**	**1**	**2**	***P***	**0**	**1**	**2**	***P***
Sex					0.337				0.957				0.605
Female		12	15	8		11	16	14		22	32	22	
Male		33	48	45		38	63	53		72	110	98	
Age(years)					**0.007**				0.409				**0.041**
<60		29	24	19		22	29	22		48	56	41	
≥60		16	39	34		27	50	45		46	86	79	
Tumor size(cm)					**0.002**				**0.012**				**<0.001**
<5		24	36	14		31	32	25		57	66	39	
≥5		21	27	39		18	47	42		37	76	81	
Location					0.852				**0.036**				0.331
UGC		28	37	30		26	56	50		54	94	79	
AEG		17	26	23		23	23	17		40	48	41	
Differentiation					0.507				0.120				0.072
Well/Moderate		13	15	10		17	21	12		30	36	22	
Poor		32	48	43		32	58	55		64	106	98	
Surgical procedure					0.613				0.104				0.340
Proxima gastrectomy		24	39	29		37	45	42		63	83	70	
Total gastrectomy		21	24	24		12	34	25		31	59	50	
PLR					**0.001**				**<0.001**				**<0.001**
<110		31	29	16		34	30	12		65	64	25	
≥110		14	34	37		15	49	55		29	78	95	
LMR					**<0.001**				**0.003**				**<0.001**
<3.25		4	25	24		4	19	24		9	45	47	
≥3.25		41	38	29		45	60	43		85	97	73	
SII					**<0.001**				**<0.001**				**<0.001**
<451		39	37	9		47	42	13		86	80	21	
≥451		6	26	44		2	37	54		8	62	99	
pTNM stage					0.104				0.101				**0.009**
I		9	4	6		11	15	5		21	19	9	
II		15	27	14		17	20	20		34	44	36	
III		21	32	33		21	44	42		39	79	75	
Adjuvant chemotherapy					0.655				0.650				0.351

Yes	21	28	28	26	46	34	41	64	63
No	24	35	25	23	33	33	53	78	57

AEG: adenocarcinoma of esophagogastric junction; UGC: upper gastric cancer; PLR: platelet-lymphocyte ratio; LMR: lymphocyte-monocyte ratio; SII:(SII=N×P/L), which was based on neutrophil (N), platelet (P) and lymphocyte (L) counts; F-NLR: combination of fibrinogen concentration and neutrophil-lymphocyte ratio.

Table S2. The correlation between fibrinogen and NLR of patients with AEG and UGC

**Patient cohort**	**Item**		**NLR**		***P***
			**<1.84(%)**	**≥1.84(%)**	
Training set	Fibrinogen	<3.09(%)	45(28.0%)	31(19.2%)	**0.030**
		≥3.09(%)	32(19.9%)	53(32.9%)	
Validation set	Fibrinogen	<3.09(%)	49(25.1%)	45(23.1%)	**<0.001**
		≥3.09(%)	34(17.4%)	67(34.4%)	
Combined set	Fibrinogen	<3.09(%)	94(26.4%)	76(21.4%)	**<0.001**
		≥3.09(%)	66(18.5%)	120(33.7%)	

NLR: neutrophil-lymphocyte ratio; AEG: adenocarcinoma of esophagogastric junction; UGC: upper gastric cancer.
